# Rapid weight gain in early life is associated with central precocious puberty in girls, not in boys - a nationwide population-based study in Korea

**DOI:** 10.3389/fendo.2023.1210995

**Published:** 2023-07-14

**Authors:** Yunsoo Choe, Jong Ho Cha, Yun Jin Kim, Jinjoo Choi, Kyeongmi Lee, Nayoung Kim, Jae Yoon Na, Seung Yang

**Affiliations:** ^1^ Department of Pediatrics, Hanyang University Guri Hospital, Guri, Republic of Korea; ^2^ Department of Pediatrics, Hanyang University Hospital, Seoul, Republic of Korea; ^3^ Biostatistical Consulting and Research Lab, Medical Research Collaborating Center, Hanyang University, Seoul, Republic of Korea; ^4^ Department of Pediatrics, Hanyang University College of Medicine, Seoul, Republic of Korea

**Keywords:** central precocious puberty, weight gain, body weight trajectory, infancy, toddlerhood

## Abstract

**Objectives:**

This study aimed to investigate the effect of rapid weight gain (RWG) on the incidence of central precocious puberty (CPP) using nationwide population-based data.

**Methods:**

A total of 253,967 children (101,841 boys and 152,126 girls) who underwent regular health consultations under the National Health Insurance Service from 2007 to 2010 were followed up until the age of 10 years for boys and 9 years for girls. We calculated differences in the weight Z-scores from 4–6 months to 9–12 months (infancy) and from 9–12 months to 18–24 months or 30–36 months (toddlerhood) using the lambda-mu-sigma method. The population was subdivided into four groups: RWGinf/tod (infancy > + 0.67 standard deviation score [SDS], toddlerhood > 0 SDS), RWGinf (infancy > + 0.67 SDS, toddlerhood < 0 SDS), RWGtod (toddlerhood > + 0.67 SDS), and control (no RWG). The diagnosis of CPP was based on the diagnostic codes of the International Classification of Diseases 10th revision and the prescription of gonadotropin-releasing hormone agonists. The cumulative risk of CPP based on age was analyzed using Kaplan–Meier survival curves, and the association between the RWG groups and CPP was assessed using multivariate logistic regression analysis.

**Results:**

CPP was diagnosed in 268 boys and 9,225 girls. For the girls, the CPP-free probability was the highest in the control group, followed by the RWGtod, RWGinf, and RWGinf/tod groups (log-rank p < 0.001). However, the incidence of CPP did not vary significantly for the boys. Compared to the control group, the other groups had a higher risk of CPP in girls (RWGinf/tod: adjusted odds ratio [aOR] 1.35, 95%, confidence interval [95% CI] 1.13–1.62; RWGinf: aOR 1.25, 95% CI 1.13–1.38; and RWGtod: aOR 1.18, 95% CI 1.09–1.28).

**Conclusions:**

This nationwide population-based study demonstrated that any RWG from birth to 3 years of age contributed to an increased risk of CPP in girls but not in boys. Girls who experienced RWG during both infancy and toddlerhood had the highest risk of developing CPP. These findings suggest that early detection and appropriate management of excessive weight gain in early life may be important for preventing CPP in girls.

## 1 Introduction

The age of pubertal onset has been reported to have declined worldwide over the past few decades (1). The incidence of precocious puberty, defined as the initiation of breast development before the age of 8 years in girls and testicular enlargement (testicular volume > 4 mL *via* palpation) before the age of 9 years in boys, has also increased (2). It is widely known that an earlier onset of puberty can result in various long-term health problems, including metabolic syndrome, type 2 diabetes (3), cardiovascular disease (4), and breast cancer (5).

A relationship between childhood obesity and early puberty has already been established (6). Several studies have revealed that the timing of puberty is closely related to rapid weight gain (RWG) during infancy, a critical period for the programming growth and development (7, 8). Salgin et al. (9) have found that even transient weight gain between 0 and 1 year of age, followed by faltering growth until 2 years of age, was associated with a younger age of menarche in girls. However, the relationship between RWG and advanced puberty is less evident for boys (10), suggesting that different mechanisms link adiposity and puberty depending on sex.

However, it remains unclear whether the effect of RWG on the onset of puberty differs depending on the timing of weight gain. Wang et al. (7) have reported that weight gain at any time from birth to 2 years has a similar effect on menarche. However, Flom et al. (11) have reported that RWG during only two periods, from 4 months to 1 year and from 1 year to 4 years, is significantly associated with the early onset of menarche. To date, the studies examining the association between RWG in infancy and the onset of puberty have been region-based cohort studies. Thus, a nationwide cohort study with multiple anthropometric measurements obtained at regular intervals is required to determine the critical period of weight gain associated with the early onset of puberty.

Hence, using nationwide birth cohort data, we aimed to investigate the association between RWG in early life and the development of precocious puberty. We divided the study population into groups according to the trajectories of weight gain from birth to 3 years of age and identified whether the weight gain patterns affected the occurrence of precocious puberty.

## 2 Methods

### 2.1 Study design and data source

We used data from the National Health Information Database (NHID), a public database on the healthcare utilization of the entire population of South Korea formed by the National Health Insurance Service (NHIS). Since 2007, the National Health Screening Program for Infants and Children (NHSPIC) has been implemented, and all children in South Korea are recommended to undergo seven health consultations by a trained pediatrician until the age of 6 years (4–6, 9–12, 18–24, 30–36, 42–48, 54–60, and 66–71 months) (12). During each consultation, anthropometric measurements, nutritional status, and developmental status are evaluated and stored in the NHID (12).

We included boys born between 2007 and 2009 and girls born between 2007 and 2010. We observed the participants until the ages of 10 years for boys and 9 years for girls as the diagnosis and treatment of precocious puberty is often delayed because of late referrals or difficulties in distinguishing the condition from slowly progressing puberty. We requested the data from individual hospital visits registered using diagnostic codes of the World Health Organization International Classification of Diseases, Tenth Revision (ICD-10) and prescription records for gonadotropin-releasing hormone (GnRH) agonists until December 31 2020. This study was conducted in accordance with the principles of the Declaration of Helsinki. The requirement for informed consent was waived in accordance with the approval of the Institutional Review Board of Hanyang University Hospital (IRB No. HYUH 2021-10-003).

**Figure 1:**
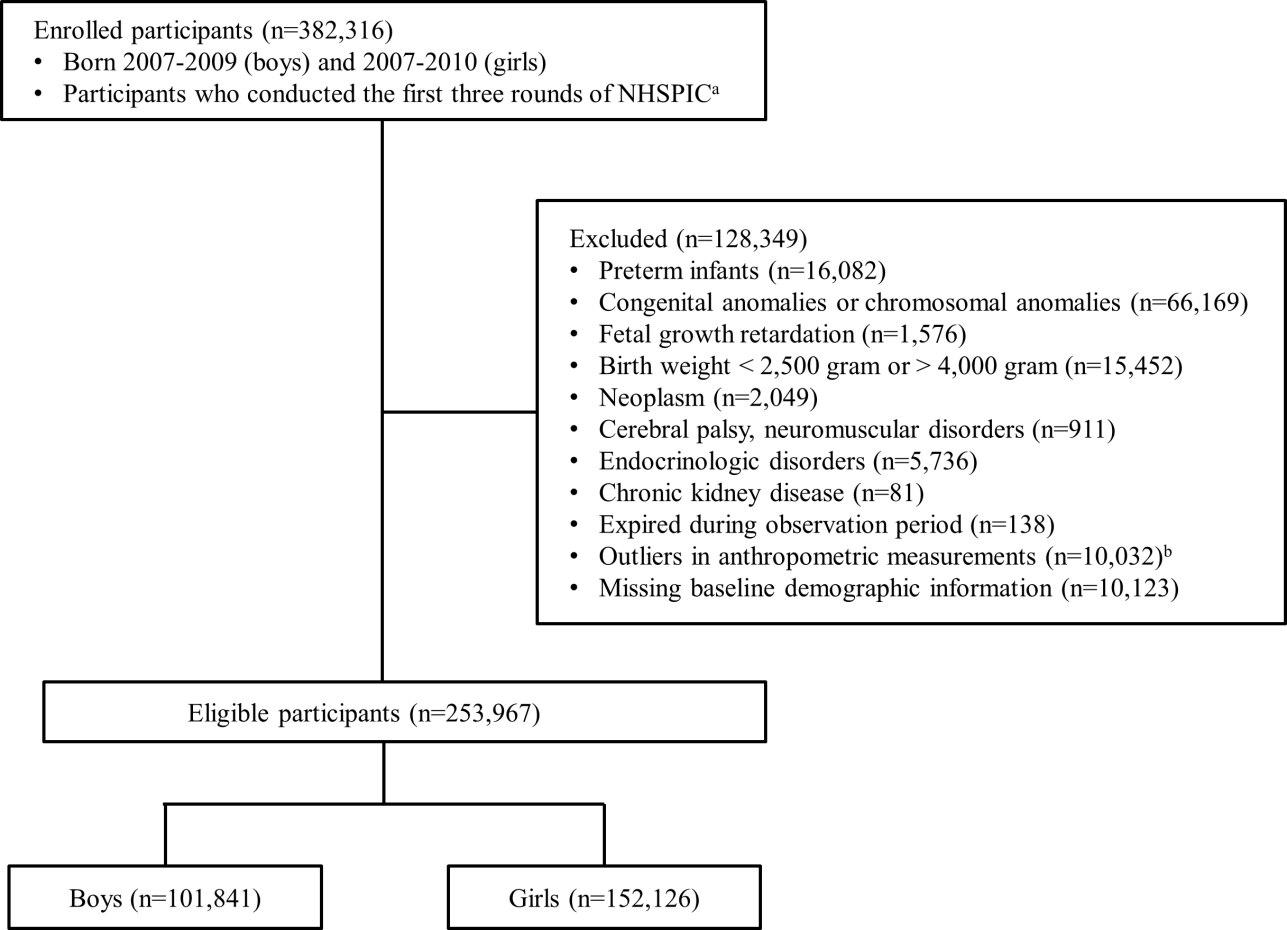
Flow diagram of study population selection. Children may have more than one cause of exclusion. NHSPIC, National Health Screening Program for Infants and Children ^a^The measurements from the 4th round (30–36 months) of the NHSPIC were used if measurements from the 3rd round were unavailable. ^b^Outliers in anthropometric measurements were defined as a Z-score > +3 or Z-score < -3 in length and weight.

To evaluate the serial changes in the anthropometric measurements of preschool children, we included 382,316 children who underwent the first three rounds of the NHSPIC. If the third round of the NHSPIC was not accessible, information from the fourth round was used. We excluded preterm-born infants and those with underlying medical conditions (n = 128,349). The detailed exclusion criteria were as follows (1): preterm infants, (2) those diagnosed with a congenital and chromosomal anomaly, (3) those diagnosed with fetal growth restriction or who were small for gestational age (SGA), (4) those with a birth weight > 4000 g or < 2500 g, (5) those diagnosed with a chronic pediatric disorder (neoplasm, ICD-10 C code; neurological disorder, ICD-10 G1, G7, and G8 code; endocrinology disorder, ICD-10 E2 code; or kidney disorder, ICD-10 N18 code), (6) those who expired during the observation period, (7) children with missing information, and (8) children who had outliers in anthropometric measurements (weight or height for age Z-score > +3 or < -3). After exclusion, the number of eligible children was 253,967 (101,841 boys and 152,126 girls), and all participants were observed until age 10 years for boys and 9 years for girls. The selection flow diagram for our study is presented in **Figure 1**.

### 2.2 Anthropometric measurements

Anthropometric measurements were obtained from NHSPIC records registered in the NHIS database. Height was measured with children in the upright position while wearing comfortable clothing, with the hips perpendicular to the central axis, heels against the footboard, and head in the Frankfurt plane. In children younger than 2 years, height was measured in the supine position. Body weight was measured using an electronic scale with children wearing simple clothing. Height was recorded to the nearest 0.1 cm and weight was recorded to the nearest 0.1 kg. Body mass index (BMI) was calculated as the weight divided by the height in meters squared. We calculated age- and sex-specific weight Z-scores through the lambda-mu-sigma (LMS) method, using the 2007 Korean National Growth Charts compiled by the Korean Centers for Disease Control (13).

### 2.3 Study population subgroups

We categorized our study population according to developmental period and occurrence of weight gain. Infancy was defined as the period between the first (4–6 months) and second (9–12 months) NHSPIC rounds, and toddlerhood was defined as the period between the second (9–12 months) and third (18–24 months) or fourth (30–36 months) NHSPIC rounds. RWG in each period was defined as a change in the weight Z-score that was greater than + 0.67 of the standard deviation score (SDS), since the 0.67 SDS corresponds to the width of each percentile band on standard growth charts (14). As a result, the study population was divided into four subgroups: 1) the “RWG during both infancy and toddlerhood (RWGinf/tod)” group was defined as the population with RWG in infancy followed by subsequent weight gain (weight Z-score change > 0 SDS) during toddlerhood, 2) the “RWG during infancy (RWGinf)” group was defined as the population with RWG only in infancy followed by subsequent decrease in weight Z-score (weight Z-score change < 0 SDS) during toddlerhood, 3) the “RWG during toddlerhood (RWGtod)” group was defined as the population with RWG only during toddlerhood, and 4) the control group was defined as the population without any RWG during infancy or toddlerhood.

### 2.4 Outcome measurement

We investigated early pubertal development using the ICD-10 diagnoses of central precocious puberty (CPP). In our study, CPP was defined as follows (1): at least one occasion of registration of the following ICD-10 codes (E22.8, central precocious puberty; E30.8, other disorders of puberty; or E30.1, precocious puberty) and (2) records of GnRH agonist (triptorelin pamoate, triptorelin acetate, or leuprolide acetate) prescription before the age of 9 years in girls and 10 years in boys. CPP is diagnosed when secondary sexual characteristics are detected before the age of 8 years in girls and 9 years in boys, along with advanced bone age and accelerated height velocity, and the GnRH stimulation test shows a peak luteinizing hormone level > 5.0 IU/L (15).

### 2.5 Statistical analysis

Baseline demographic characteristics between the groups were compared using the chi-square test or Fisher’s exact test for categorical variables and the Kruskal–Wallis test for continuous variables, after the Anderson–Darling normality test. Subsequently, a *post-hoc* analysis using the Bonferroni correction was performed. The cumulative risk of CPP based on age was presented using Kaplan–Meier survival curves, and all four subgroups were compared using the log-rank test. To identify whether the risk of CPP differed according to the population subgroup, a multivariate logistic regression test was performed and the adjusted odds ratios (aORs) and 95% confidence intervals (CIs), using the control group as a reference, were presented. For covariates, sex, socioeconomic status, residence, birth weight (per 100 g strata), and primary milk feeding type (breastfeeding only, formula feeding only, or mixed feeding) were selected. Socioeconomic status was based on health insurance premiums, which are proportional to household incomes, and categorized into quartiles. Birth weight and milk feeding type were based on parent-answered questionnaires during the first NHSPIC round. Statistical analyses were performed using the SAS version 9.4 (SAS Institute Inc., Cary, NC, USA).

## 3 Results

### 3.1 Characteristics of the study population

The baseline demographic characteristics of the study population are summarized in **Table 1**. Overall, 2,366 children (0.9%) were classified into the RWGinf/tod group, 8,851 (3.5%) into the RWGinf group, 16,470 (6.5%) into the RWGtod group, and 226,280 (89.1%) into the control group. Females were predominant in all subgroups as well as in the total population (n = 152,126, 59.9%). Birth weight was the lowest in the RWGinf/tod group, followed by the RWGinf, RWGtod, and control groups, although the observed differences were not substantial.

**Table 1:** Demographic characteristics of the study population.

	RWGinf/tod (n = 2,366)	RWGinf (n = 8,851)	RWGtod (n = 16,470)	Control (n = 226,280)	*p* value ^†^
Birth weight, kg	3.16 ± 0.34	3.19 ± 0.34	3.21 ± 0.34	3.23 ± 0.34	< 0.001 ^a,b,c,d,e,f^
Sex					< 0.001 ^a,b,c,e,f^
Male	763 (32.3)	3,110 (35.1)	5,877 (35.7)	92,091 (40.7)	
Female	1,603 (67.8)	5,741 (64.9)	10,593 (64.3)	134,189 (59.3)	
Primary milk feeding type					< 0.001 ^b,c,d,e,f^
Breastfeeding only	834 (35.3)	3,031 (34.2)	8,882 (53.9)	111,798 (49.4)	
Formula feeding only	897 (37.9)	3,505 (39.6)	4,668 (28.3)	71,720 (31.7)	
Mixed feeding	635 (26.8)	2,315 (26.2)	2,920 (17.7)	42,762 (18.9)	
Socioeconomic status^*^					< 0.001 ^c,d,e,f^
1^st^ quartile	292 (12.3)	979 (11.1)	1,832 (11.1)	24,109 (10.7)	
2^nd^ quartile	640 (27.1)	2,430 (27.5)	4,246 (25.8)	57,058 (25.2)	
3^rd^ quartile	950 (40.2)	3,596 (40.6)	6,787 (41.2)	93,131 (41.2)	
4^th^ quartile	484 (20.5)	1,846 (20.9)	3,605 (21.9)	51,982 (23.0)	
Residence					< 0.001 ^a,e,f^
Urban area	1,175 (49.7)	4,161(47.0)	7,876 (47.8)	110,661 (48.9)	
Rural area	1,191 (50.3)	4,690(53.0)	8,594 (52.2)	115,619 (51.1)	

RWGinf/tod, rapid weight gain during both infancy and toddlerhood (weight Z-score change> +0.67 during infancy, weight Z-score change > 0 during toddlerhood); RWGinf, rapid weight gain during infancy (weight Z-score change > +0.67 during infancy, weight Z-score change < 0 during toddlerhood); RWGtod, rapid weight gain during toddlerhood (weight Z-score change < + 0.67 during infancy, weight Z-score change > + 0.67 during toddlerhood).

* Socioeconomic status was based on the amount of health insurance premium. Income status was categorized into quartiles.

Data are expressed as mean ± standard deviation for continuous variables and percentages (%) for categorical variables.

^†^ post-hoc p value is expressed as follows: a (RWGinf/tod vs. RWGinf), b (RWGinf/tod vs. RWGtod), c (RWGinf/tod vs. Control), d (RWGinf vs. RWGtod), e (RWGinf vs. Control), and f (RWGtod vs. Control).

**Figure 2:**
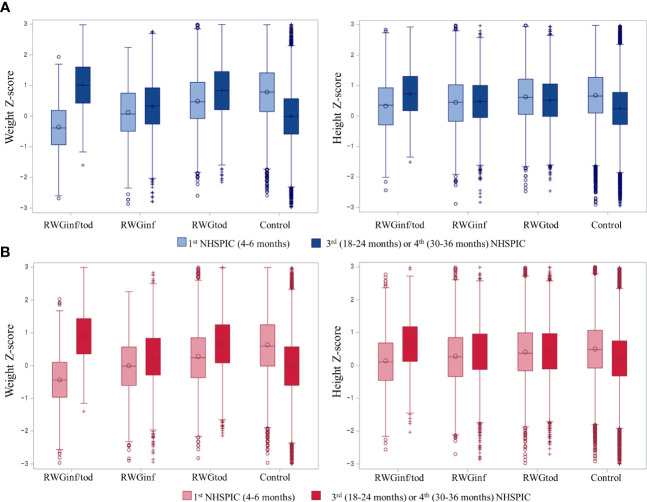
Box and whisker plot of the Z-scores of the weight and height per population subgroup in **(A)** boys and **(B)** girls. The left whisker plot represents anthropometric measurements from the 1st round (4–6 months) of NHSPIC, and the right whisker plot represents anthropometric measurements from the 3rd (18–24 months) or 4th (30–36 months) rounds of NHSPIC. RWGinf/tod, rapid weight gain during both infancy and toddlerhood; RWGinf, rapid weight gain during infancy; RWGtod, rapid weight gain during toddlerhood; NHSPIC, National Health Screening Program for Infants and Children.

The weight and height Z-score changes between the 1st and 3rd or 4th NHSPIC rounds, according to subgroup population, are presented in **Figure 2**. For the anthropometric measurements obtained at 4–6 months of age, the RWGinf/tod group had the lowest length and weight Z-scores, followed by those of the RWGinf, RWGtod, and control groups.

### 3.2 Incidence of CPP and comparison of demographic characteristics

CPP was diagnosed in 268 boys and 9,225 girls during the study period. The incidence rate of CPP was 26.3 persons/100,000 person-years in boys and 673.8 persons/100,000 person-years in girls. The demographic and socioeconomic characteristics according to CPP diagnosis are presented in **Table 2**. The median age of diagnosis was 9.6 (interquartile range [IQR]: 9.0–9.9) years in boys and 8.2 (IQR: 7.6–8.7) years in girls. Girls with CPP had a slightly lower birth weight compared to those without CPP (3.17 ± 0.33 kg vs. 3.20 ± 0.33 kg, p < 0.001). Similarly, although statistically significant, the differences in socioeconomic status between girls with and without CPP were not pronounced. However, it is noteworthy that the proportion of exclusive breastfeeding was significantly higher among girls without CPP (50.9% vs. 46.8%, p < 0.001), suggesting a notable protective effect of breastfeeding against CPP. In contrast, there were no significant differences in birth weight, primary milk feeding type, socioeconomic status, or residence between boys with and without CPP.

**Table 2:** Comparison between central precocious puberty-diagnosed and non-diagnosed groups.

	Boys			Girls		
	CPP (+) (n = 268)	CPP (-) (n = 101,573)	*p* value	CPP (+) (n = 9,225)	CPP (-) (n = 142,901)	*p* value
Median age of diagnosis, yr	9.6 (9.0–9.9)			8.2 (7.6-8.7)		
Birth weight, kg	3.23 ± 0.33	3.28 ± 0.34	0.13	3.17 ± 0.33	3.20 ± 0.33	< 0.001
Primary milk feeding type			0.11			< 0.001
Breastfeeding only	113 (42.2)	47,450 (46.7)		4,317 (46.8)	72,665 (50.9)	
Formula feeding only	105 (39.2)	33,625 (33.1)		3,056 (33.1)	44,004 (30.8)	
Mixed feeding	50 (18.7)	20,498 (20.2)		1,852 (20.1)	26,232 (18.4)	
Socioeconomic status^*^			0.30			< 0.01
1^st^ quartile	25 (9.3)	10,866 (10.7)		978 (10.6)	15,343 (10.7)	
2^nd^ quartile	63 (23.5)	26,184 (25.8)		2,187 (23.7)	35,940 (25.2)	
3^rd^ quartile	107 (39.9)	41,588 (40.9)		3,926 (42.6)	58,843 (41.2)	
4^th^ quartile	73 (27.2)	22,935 (22.6)		2,134 (23.1)	32,775 (22.9)	
Residence			0.58			0.46
Urban area	138 (51.5)	50,547 (49.8)		4,473 (48.5)	68,715 (48.1)	
Rural area	130 (48.5)	51,026 (50.2)		4,752 (51.5)	74,186 (51.9)	

CPP, central precocious puberty.

^*^ Socioeconomic status was based on the amount of health insurance premium. Income status was categorized into quartiles.

Data are expressed as medians (25^th^–75^th^ percentile) or mean ± standard deviation for continuous variables and percentages (%) for categorical variables.

### 3.3 Risk of CPP according to the weight gain subgroup

**Figure 3:**
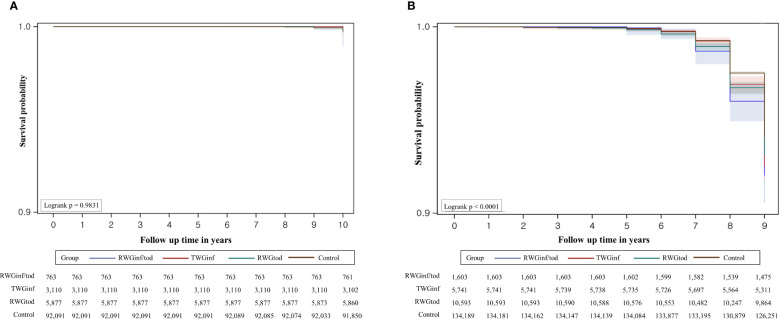
Overall survival estimates with 95% confidence limits and the number of subjects at risk in **(A)** boys and **(B)** girls. RWGinf/tod, rapid weight gain during both infancy and toddlerhood; RWGinf, rapid weight gain during infancy; RWGtod, rapid weight gain during toddlerhood.

The Kaplan–Meier survival curves for CPP based on the age at diagnosis are presented in **Figure 3**. For boys, the incidence rate of CPP did not vary according to the subgroup. For girls, the CPP-free probability was highest in the control group, followed by the RWGtod, RWGinf, and RWGinf/tod groups (log-rank p < 0.001). In girls, notable differences between the groups start to emerge around the ages of 7 to 7.99 years, and these differences become particularly prominent between the ages of 8 and 8.99 years. On the contrary, in boys, the diagnosis of CPP occurs mostly between the ages of 9 and 10 years although it is not statistically significant.

According to the multivariate logistic regression analysis results summarized in **Table 3**, the effect of weight gain at an early age differed according to sex. For boys, weight gain at an early age was not associated with CPP. Boys with higher birth weights had a lower risk of CPP in the multivariate analysis (aOR 0.69, 95% CI 0.49–0.97). For girls, the risk of CPP was the highest in the RWGinf/tod group (aOR 1.35, 95% CI 1.13–1.62), followed by the RWGinf (aOR 1.25, 95% CI 1.13–1.38), RWGtod (aOR 1.18, 95% CI 1.09–1.28), and control groups. Similar to the finding in boys, higher birth weight had a significant protective effect on CPP (aOR 0.80, 95% CI 0.75–0.85) in girls. Meanwhile, formula feeding (aOR 1.16, 95% CI 1.11–1.22) and mixed feeding (aOR 1.18, 95% CI 1.12–1.25) were significantly associated with an increased risk of CPP.

**Table 3:** Multivariate logistic regression analysis showing an association between rapid weight gain during early life and pubertal development.

	Boys			Girls		
	aOR	95% CI	*p* value	aOR	95% CI	*p* value
Population subgroup						
RWGinf/tod	0.95	0.24-3.83	0.94	1.35	1.13-1.62	< 0.01
RWGinf	0.94	0.47-1.91	0.87	1.25	1.13-1.38	< 0.001
RWGtod	1.08	0.66-1.77	0.75	1.18	1.09-1.28	< 0.001
Control	Reference			Reference		
Birth weight, kg	0.69	0.49-0.97	0.03	0.80	0.75-0.85	< 0.001
Primary milk feeding type						
Breastfeeding only	Reference			Reference		
Formula feeding only	1.30	0.999-1.97	0.051	1.16	1.11-1.22	< 0.001
Mixed feeding	1.02	0.73-1.42	0.91	1.18	1.12-1.25	< 0.001
Socioeconomic status^*^						
1^st^ quartile	Reference			Reference		
2^nd^ quartile	1.02	0.65-1.61	0.92	0.95	0.88-1.03	0.22
3^rd^ quartile	1.09	0.71-1.67	0.69	1.05	0.98-1.13	0.16
4^th^ quartile	1.37	0.87-2.14	0.17	1.03	0.95-1.11	0.53
Residence						
Urban area	Reference			Reference		
Rural area	0.97	0.76-1.23	0.78	0.98	0.94-1.02	0.40

RWGinf/tod, rapid weight gain during both infancy and toddlerhood(weight Z-score change > +0.67 during infancy, weight Z-score change > 0 during toddlerhood); RWGinf, rapid weight gain during infancy (weight Z-score change > +0.67 during infancy, weight Z-score change < 0 during toddlerhood); RWGtod, rapid weight gain during toddlerhood (weight Z-score change < +0.67 during infancy, weight Z-score change > +0.67 during toddlerhood); aOR, adjusted odds ratio; CI, confidence interval.

^*^ Socioeconomic status was based on the amount of health insurance premium. Income status was categorized into quartiles.

## 4 Discussion

Using nationwide prospective birth cohort data, we found that girls with RWG in early life had a higher CPP risk. Specifically, excessive weight gain during any period before 3 years of age was significantly associated with CPP, and the effect was the most prominent from 4–6 months to 9-12 months. However, the association between RWG and CPP was not significant in boys.

Our study found that the overall incidence rate of CPP in South Korea was 673.8 persons/100,000 person-years in girls, which was approximately 25 times that in boys, who had an overall incidence of 26.3 persons/100,000 person-years. This incidence rate was significantly higher than those observed in previous nationwide Korean studies (16, 17), albeit considering the steeply rising trend. The incidence rate of CPP was reported to be 15.3 per 100,000 girls and 0.6 per 100,000 boys from 2004 to 2010 and 262.8 per 100,000 girls and 7.0 per 100,000 boys from 2008 to 2014 (16, 17). This large discrepancy between the estimated incidence in our study and those reported in other studies may originate from methodological differences. We excluded children who were born preterm, had chronic disorders (i.e., cerebral palsy or other congenital anomalies), and had disorders that can induce growth problems (i.e., chronic kidney disease or neoplasms) to investigate the association of RWG in early life with pubertal development in healthy Korean children. In addition, selection bias may have occurred because the study included only individuals who underwent at least three rounds of the NHSPIC, whose parents may have been more interested in the pubertal developmental status of their child. Therefore, our results may have overestimated the incidence of CPP.

In this study, RWG in early life increased the risk of precocious puberty in girls, but not in boys. In girls, even a transient increase in weight during infancy increased the risk of CPP, and the size of this effect was greater during infancy than during toddlerhood. Girls who experienced RWG during both infancy and toddlerhood had the highest risk of CPP. Sex-specific dimorphism in the effect of weight gain on the onset of pubertal development has been previously reported (18). However, the mechanisms underlying the varied effects of infancy weight gain on puberty remain unclear. RWG during infancy may increase leptin levels, which have a permissive role in the activation of GnRH neurosecretion in the hypothalamus (6). During puberty, girls typically have consistently elevated levels of leptin, whereas boys experience initially elevated levels of leptin that lower in late puberty (19). Adrenal androgen is another important hormone linked to RWG in infancy and early puberty. Higher levels of adrenal androgens, which are linked to early puberty, as observed in congenital adrenal hyperplasia, have been reported in 8-year-old children with low birth weight and RWG during infancy (20). In addition, RWG-induced insulin resistance and hyperinsulinemia lower the levels of sex hormone-binding globulin, resulting in the increased concentration and bioavailability of free sex hormones (21). An increase in insulin-like growth factor (IGF-1) concentration following RWG in early life also influences the age of pubertal onset (22). During puberty, girls typically have higher levels of insulin, IGF-1, and leptin than boys. This difference in hormone levels may mediate the effect of early weight gain in advancing the age of pubertal onset differently depending on sex.

We found that low birth weight was a significant predictor for the occurrence of CPP in both boys and girls from the multivariate analysis, although the differences were not quite remarkable. It has been suggested that girls who weigh light at birth experience menarche at an early age (23–25). This may be explained by the additive effect of catch-up growth following low birth weight, which may accelerate puberty (25). In this study, we analyzed both birth weight and weight gain trajectories in early life and found that in girls, both factors were independent risk factors for CPP, whereas in boys, only low birth weight had a significant effect. It is plausible that the combination of insufficient intrauterine growth and subsequent catch-up has a synergistic effect on pubertal development, particularly in girls. Furthermore, the validity of our study was increased by the exclusion of preterm, SGA, and low-birth-weight infants weighing < 2.5 kg, which are factors known to be associated with early puberty. This relationship between decelerated fetal growth and early puberty can be explained by the Developmental Origins of Health and Disease theory (26). According to this theory, the prenatal and postnatal nutritional environments affect the onset and progression of pubertal development. Thus, when a female fetus experiences nutritional limitation *in utero*, certain epigenetic programming mechanisms may occur to reduce the age of menarche and modify its reproductive capabilities (27). Our study results add credence to the concept that, aside from other well-known risk factors such as obesity, low birth weight can contribute to advancing the onset of puberty.

The strengths of this study included the collection of nationwide records of early childhood anthropometric measurements of children reliably diagnosed with CPP using a GnRH stimulation test and treated with GnRH agonists using health insurance claim data. This method was more objective and reliable than using subjective markers of puberty, such as the Tanner stage, or relying on memory recall in describing pubertal progression. Particularly in boys, it can be difficult to determine the timing of puberty since it begins with testicular enlargement. We prevented these limitations by classifying CPP based on the NHIS data. Additionally, we adjusted for potential confounders, such as socioeconomic status or milk-feeding type, that may affect the age of puberty. However, this study had some limitations. First, a high-nutrition diet and an overweight or obese status in the prepubertal period, which are known to increase the risk of CPP, were not included in the analysis. Second, other information regarding pubertal development (i.e., maternal age of menarche), which may confound our findings, was not included in the analysis because of the limitations of the NHIS data. Furthermore, the potential for bias exists since we only included cases of CPP treated with GnRH agonist which may limit the generalization of our study to reflect the true incidence of CPP. On contrary, given the markedly steep increase in CPP incidence observed in South Korea, it is essential to consider the possibility of overtreatment including mild cases, within the clinical context. Despite these limitations, this study is valuable because it is the first study to evaluate the association between early RWG and CPP using nationwide cohort data.

In conclusion, we observed that RWG during any period from birth to 3 years of age was associated with CPP in girls but not in boys. Girls who experienced excessive weight gain during both infancy and toddlerhood had the highest risk of developing CPP. Our findings provide evidence supporting the negative effects of increased adiposity in early life. Early detection of RWG during infancy and toddlerhood enables the application of prompt interventions to avoid early puberty and early puberty-related health problems later in life.

## Data availability statement

The original contributions presented in the study are included in the article/supplementary material. Further inquiries can be directed to the corresponding authors.

## Ethics statement

The studies involving human participants were reviewed and approved by Institutional Review Board of Hanyang University Hospital. Written informed consent from the participants’ legal guardian/next of kin was not required to participate in this study in accordance with the national legislation and the institutional requirements.

## Author contributions

JHC, YJK, JYN, and SY conceived and designed the study. JHC and YJK analyzed the data. YC, JHC, YJK, JC, KL, NK, JYN, and SY interpreted the data. YC and JHC wrote the first draft of this manuscript. YC, JHC, YJK, JC, KL, NK, JYN, and SY critically reviewed and edited the manuscript. All authors contributed to the manuscript and approved the submitted version.
